# Experimental Study on Influence of Curing Time on Strength Behavior of SLA-Printed Samples Loaded with Different Strain Rates

**DOI:** 10.3390/ma13245825

**Published:** 2020-12-21

**Authors:** Danuta Miedzińska, Roman Gieleta, Arkadiusz Popławski

**Affiliations:** Faculty of Mechanical Engineering, Military University of Technology, Kaliskiego 2 St., 00-908 Warsaw, Poland; roman.gieleta@wat.edu.pl (R.G.); arkadiusz.poplawski@wat.edu.pl (A.P.)

**Keywords:** stereolithography, strain rate, photocurable resin, mechanical properties, additive manufacturing

## Abstract

Stereolithography (SLA) is an additive manufacturing process based on the photocuring of resins with the use of UV light. The printed samples can be used not only for the visualization of structures, but also to develop elements of real constructions. In the study, SLA-printed samples made of Formlabs’ Durable Resin were tested in static, dynamic, and Hopkinson’s bar tests. The recommended UV and heat curing time for this resin is 60 min for each process. For the tests, 5-minute and 30-min curing times were also considered. The obtained stress-strain curves were compared. The resin showed a difference in response to the strain rate effect and a curing time influence was noticed. For the static tests, the post-curing time had the greatest effect with a very small standard deviation. For the dynamic tests, similar dependencies were observed but with a greater standard deviation. The tests at very high strain rates were associated with a much greater level of difficulty in execution, recording, and signal analyzing, and the influence of the exposure time on the mechanical properties was not straightforward. The tested resin showed strengthening with increases in the strain rate as well as in the curing time.

## 1. Introduction

**Figure 1:**
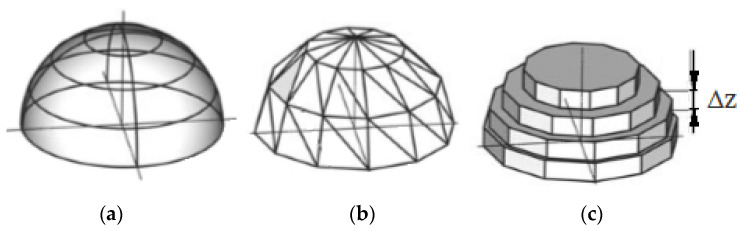
Scheme of the stereolithography (SLA) printing process: (**a**) CAD model, (**b**) STL model, and (**c**) division into slices. Δz—layer thickness.

**Figure 2:**
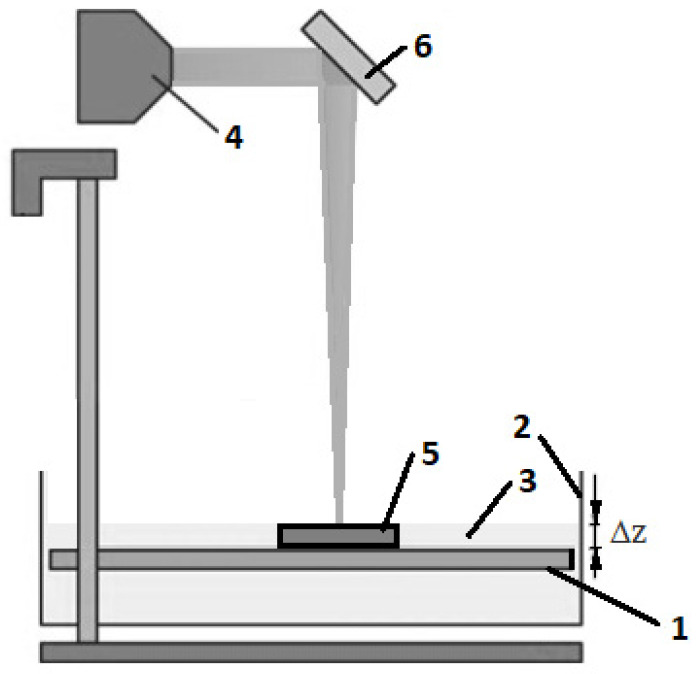
SLA printer scheme: (**1**) moving platform, (**2**) resin tank, (**3**) liquid polymer, (**4**) ultraviolet (UV) laser, (**5**) printed object, (**6**) mirror. Δz—layer thickness.

Stereolithography (SLA) is one of the earliest additive manufacturing processes to be used [1,2]. The method is based on printing using special photocurable resin. The resin is crosslinked under ultraviolet (UV) light exposure, called photopolymerization [3]. Special printers were designed to carry out the SLA process. The scheme of such a 3D printer is presented in Figure 1. The first step of SLA-printed object creation is the development of a CAD model (Figure 1a), which is converted to an STL format (Figure 1b). Special software divides the model into slices (Figure 1c). The input file is used to print the sample slice by slice. The SLA printer has a moving platform (marked 1 in Figure 2), and this platform carries the tank (marked 2 in Figure 2) filled with a liquid polymeric resin (marked 3 in Figure 2). The UV laser (marked 4 in Figure 2) cures the desired pattern of the sample layer (marked 5 in Figure 2) on the surface of the resin through the mirror (marked 6 in Figure 2). Then the platform (marked 1 in Figure 2) moves, and the curing process is repeated. The layer thickness is Δz (Figure 1 and Figure 2) [4].

Some examples of the mechanical properties of SLA-printed materials can be found in the literature. Zhao et al. [5] synthesized a polyurethane acrylate compounded with epoxy acrylate, isobornyl acrylate, and a radical photo-initiator. The fold-deploy test and shape memory cycle measurements were carried out and proved the excellent shape memory performance of the printed objects, including a high shape recovery rate, high shape fixity, and excellent endurance. A tensile test at 70 °C was carried out and the printed objects showed high strength and good toughness.

The effects of build orientation on tensile strength for stereolithography-manufactured ASTM D-638 type I specimens were presented by Quintana et al. [6]. In the paper, a statistical design of experiments (DOE) approach was used to determine whether specific build orientation parameters impacted the mechanical strength of stereolithography (SLA)-fabricated parts.

A study of the influence of supports on the tensile strength of SLA-printed samples was reported by Kazemi and Rahimi [7]. In the paper, it was proven that supporting structures influence the tensile strength by increasing the roughness of the sample surface. In addition, the authors concluded that the strength parameter of samples supported symmetrically was slightly lower than in samples supported unsymmetrically.

Chantarapanich et al. studied the influence of build orientation and UV exposure time on the strength behavior of Watershed 11122, which is a commercially available photocurable epoxy resin [8]. The analysis showed that the mechanical behavior of samples differed in a negligible way among sub-build orientations. In addition, there were small variations in mechanical properties between the main build orientations. The strength of samples increased with increasing UV exposure time. This phenomenon was observed for 0–4 h.

Belter and Dollar presented a method for improving the mechanical properties of 3D-printed thermoplastic samples by developing voids in the samples and filling them with high-strength resins [9].

The basic tensile strength and elastic modulus of printed components produced with FDM (fused deposition modeling) and SLA printers were investigated by Szykiedans and Credo [10]. The tests were carried out using ABS (acrylonitrile butadiene styrene), fiberglass-reinforced polyethylene terephthalate glycol (Z-Glass), and a Nobel printer photo-resistive resin. The results showed some distinctions between the tensile modulus of 3D prints and their base materials.

Sakly et al. used Al-based quasicrystalline alloys to develop a new UV-curable resin reinforced by these metal particles [11]. 3D composite parts were built by SLA and showed improved mechanical properties in comparison to the unfilled resin (higher hardness, reduced wear loss, and lower friction coefficient).

Dar et al. studied the mechanical properties of SLA-printed lattice structures, such as BCC (body-centered cubic system). They carried out both experimental and finite element analyses. For material model development, quasi-static tensile tests were performed [12].

Fry et al. presented a study on strength behavior and damage of samples printed from clear and tough resins produced by Formlabs. They carried out static compression tests until the complete fracturing of samples [13].

Li et al. synthesized core-shell nanoparticles for SLA printing, which showed improved mechanical properties in static tensile tests and were assessed as similar to ABS (acrylonitrile butadiene styrene) [14]. Another static tensile test was presented by Patel et al. in which UV-curable elastomers were tested and showed 1100% elongation [15]. The tensile strength of 3D-printed samples was also shown by Sagias et al. [16], Dizon et al. [17], and Maso and Cosmi [18], and compression strength was studied by Mercado-Colmenero et al. [19] and Casavola et al. [20]. Uniaxial tensile tests were carried out on standard SLA-printed specimens to obtain their elastic modulus and ultimate tensile strength in order to develop the constitutive material model by Wang et al. [21].

The approach by Puebla et al. and Dulieu-Barton and Fulton was directed at investigating how the effects of aging, pre-conditioning, and build orientation influenced the mechanical properties of test samples fabricated using SLA and a commercially available resin [22,23]. The effects of the orientation of layers on the peak stress, elongation at break, and Young’s modulus (the modulus of elasticity) of SLA polymeric samples were studied by Layton and Rosen [24]. The influence of laser power in the manufacturing process of SOMOS 7110 photosensitive resin was investigated by Salmoria et al. [25]. The optimization of curing parameters due to compression and tensile strengths was presented as a designed process by Chockalingam et al. [26], and the influence of layer thickness on mechanical properties was also considered by Chockalingam, Jawahar, and Chandrasekhar [27].

Basic mechanical properties have also been reported by resin producers [28]. The data showed that post-curing at higher temperatures resulted in a shorter time to full cure. In addition, higher temperatures improved mechanical properties.

It must also be mentioned that the authors of the presented paper have also published a study that experimentally tested tough and clear resins (produced by Formlabs) via the static compression test and dynamic Hopkinson’s bar loading [29]. The tested materials showed visible differences when responding to different strain rate loadings.

## 2. Aim of Research

Based on the literature review presented above, it can be considered that there has been no study on the influence of the strain rate and curing time on the mechanical behavior of SLA-printed samples. In the presented paper, experimental research on Durable Resin, produced by Formlabs, is shown. The resin is recommended by the manufacturer for parts requiring high impact strength, prototyping parts that will be made out of polypropylene or high density polyethylene (HDPE), and parts that require a low-friction surface, such as ball joints, parts that are both rigid and flexible, snap-fit parts, and flexures.

On the basis of the manufacturer’s recommendations, it was decided to test the samples with static, dynamic, and Hopkinson’s bar tests to check the resin’s behavior under different strain rates.

In addition, the manufacturer recommends a curing time of 60 min for UV and heat exposure. To check the influence of this parameter, the curing time was also lowered to 5 and 30 min. These samples were also tested with the strain rates described above.

The presented work is important for the application of SLA-printed materials in real constructions, e.g., as elements of military or safety structures, where the strain rate effect on material properties can be significant. The most effective approach in designing such structures is coupling experimental and computational analyses. The observation of change in the strength behavior of these new materials loaded with different strain rates can lead to proper material model selection and to the development of safe and reliable constructions.

## 3. Research Description

The analysed material is a photocurable resin—Durable Resin—produced by Formlabs. The producer declares that it is impact resistant and highly wear resistant, which means it is capable of extreme deformation before breaking. Its properties should be similar to polypropylene (PP) or high density polyethylene (HDPE). The resin is composed of a mixture of methacrylic acid esters, photo-initiators, proprietary pigment, and additive packages [30]. The detailed chemical composition as well as the polymerization groups are not allowed to be given.

The photopolymerization process applied in SLA printing is well known. It is based on the assumption that the photocurable resins have short carbon chains (from 1 carbon to a few thousand carbons). They have all components of the final plastic (the photopolymerized material) but are not fully polymerized. The UV exposition causes the chains to become longer to create the solid part.

**Figure 3:**
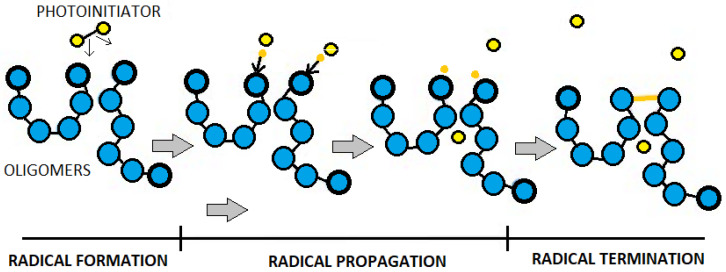
Scheme of photopolymerization of the stereolithography (SLA) resin.

The monomer and oligomer chains in the SLA resin are built of active groups at their ends. In the resin exposed to UV light, the photo-initiator molecule breaks down into two parts, and the bond holding it together becomes two very reactive radicals. The photo-initiator transfers the reactive radicals to the active groups on the monomers and oligomer chains, which, in turn, react with other active groups, forming longer chains [30]. The process is very short and lasts milliseconds. The photopolymerization process was schematically presented in Figure 3.

**Figure 4:**
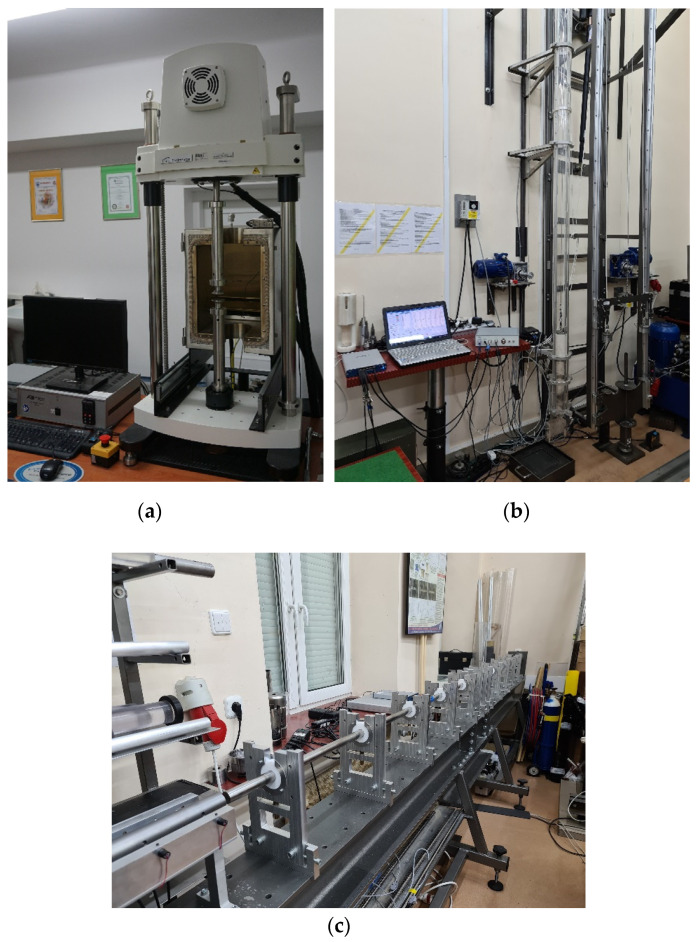
Test equipment: (**a**) ElectroForce 3330 Series II Axial, (**b**) impact hammer, and (**c**) Split Hopkinson’s Pressure Bar (SHPB).

To assess the strain rate effect on durable resin, three different tests and machines were applied:

- Static test—the ElectroForce 3330 Series II Axial (Figure 4a) achieved strain rates of 0.001 1/s and 0.84 1/s,
- Dynamic test—the impact hammer (own construction, Figure 4b) achieved strain rates of 500 1/s and 1000 1/s,
- Hopkinson’s bar test—the Split Hopkinson’s Pressure Bar (SHPB, own construction, Figure 4c) achieved strain rates of 4317, 5157, 6281, and 7994 1/s.

The ElectroForce 3330 Series II (TA Instruments, New Castle, DE, USA) testing stage is dedicated to carry out static tensile, compression, and bending tests with a load range of up to 3000 N. The traverse drive is electric, which allows for stepless speed regulation from 0.02 μm/s to 1 m/s.

This impact machine is a drop hammer (own construction, MUT, Warsaw, Poland) and can achieve energy up to 1 kJ. The force measurement is realized using a specialized dynamometer for impact loads, the displacement measurement by using a laser displacement sensor, and the speed measurement by using laser barriers. A computer system for the control, acquisition, and processing of measurement data is also applied.

The high strain rate testing stage (the SHPB—own construction, MUT, Warsaw, Poland) measuring rod material was V720 maraging steel. The length of the measuring rods was 2000 mm, the diameter of the measuring rods was between 10 and 60 mm, and the pressure in the tank went up to 12 bar. The machine has programmatic pressure regulation in the tank with a proportional valve. The projectile velocity measurement is carried out using laser barriers. The programs for station control and the acquisition and processing of measurement signals are implemented in the stage.

The strain rate value was achieved in the tests by the displacement speed of the strength machine clamps, the impact velocity of the hammer head (1.5 and 3 m/s for a sample height of 3 mm), and, in the SHPB test, the different sample heights (1, 1.5, 2, and 2.5 mm), which had the same projectile propulsion pressure.

The cylindrical samples were printed using a Formlabs Form 2 SLA printer (Formlabs Inc., Somerville, MA, USA). The dimensions for each test with the achieved strain rates are presented in Table 1.

The process of sample preparation was as follows.

**Figure 5:**
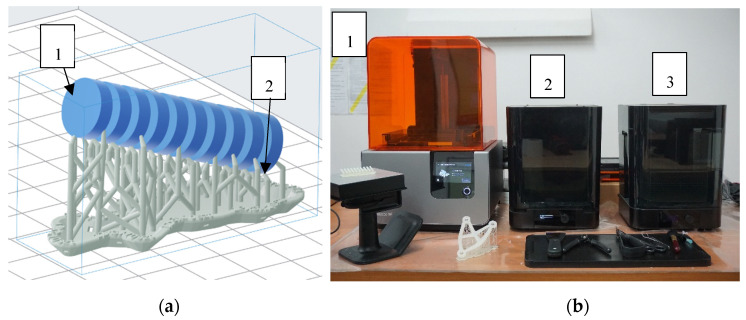
Sample preparation: (**a**) samples designed in PreForm software (1—samples, 2—supports), (**b**) Formlabs setup: 1—SLA printer, 2—curing chamber (UV and heat exposition), 3—cleaning chamber for isopropanol bath.

- Step 1: the geometry of the samples was designed in Solid Works (Version 28, 2020) and transformed to PreForm (Version 3.3.2) software to prepare the printing process. The supports were automatically set in the model (Figure 5a).
- Step 2: the samples were printed using Formlabs Form 2 SLA printer, the layer height was 0.05 mm in accordance with the manufacturer’s guidelines to achieve the best strength parameters [13] (Figure 5b).
- Step 3: the supports were removed mechanically by using a knife, and the samples were cleaned using an isopropanol bath (Figure 5b).
- Step 4: the samples were exposed to UV radiation and annealed in a thermal chamber at 60 °C for periods of 5, 30, and 60 min for each process.

The curing time selection was based on the resin manufacturer’s guidelines [13], which state that the maximum strength is achieved after 1 h of UV and heat exposure. It was decided to shorten both of those times to 50% and 8.3% to check the influence of those coupled factors. The time change was significant because the purpose was to check the influence of curing parameters instead of finding the time after which the polymerization and hardening process is finished.

The total number of samples tested, taking into account the three sample states of 5, 30, and 60 min, were 18 samples for static tests, 30 samples for dynamic tests, and 120 samples for SHPB tests.

## 4. Results and Discussion

The results are presented as stress-strain curves. Engineering stress and strain were assumed, which, for the static and dynamic tests, meant that the values were calculated on the basis of the initial dimensions of the samples. For the SHPB test, the values were calculated with the following equations [31].

- engineering stress:

(1) $$\sigma_{N}\left(t\right)=\frac{EA}{A_{S}}\varepsilon_{t}\left(t\right)$$

- engineering strain:

(2) $$\varepsilon_{N}\left(t\right)=-2\frac{C_{0}}{L}\int_{0}^{t}\varepsilon_{r}\left(t\right)dt$$

where *E* is the Young’s modulus of the rod material, *A* is the cross-sectional area of the rods, *A_S_* is the cross-sectional area of the sample, *ε_t_(t)* and *ε_r_(t)* are the impulses of transmitted and reflected waves, respectively, *C_0_* is a speed of the elastic wave in the measuring rods, and *L* is the length of the sample.

**Figure 6:**
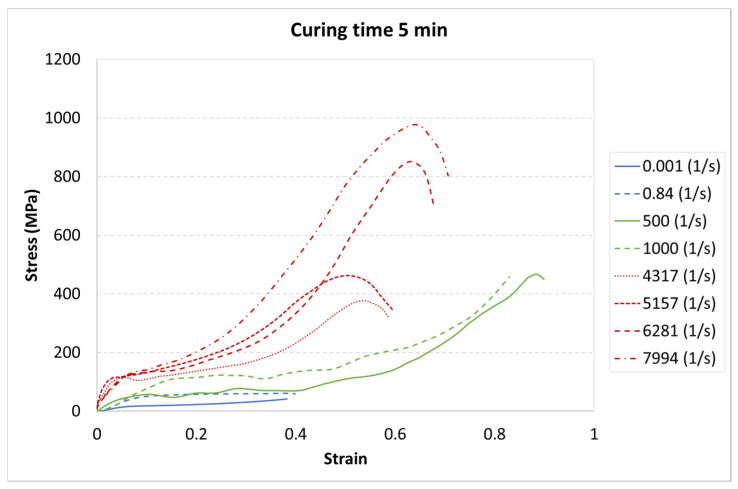
Results of durable resin tested under various strain rates for a curing time of 5 min for ultraviolet (UV) and heat exposure.

**Figure 7:**
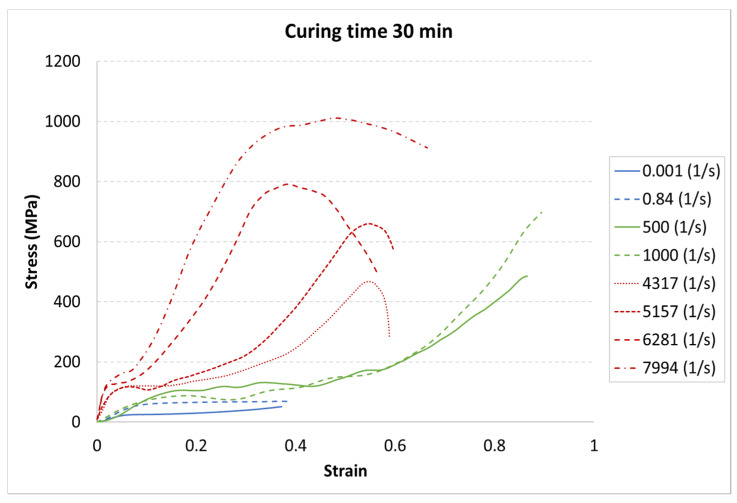
Results of durable resin tested under various strain rates for a curing time of 30 min for UV and heat exposure.

**Figure 8:**
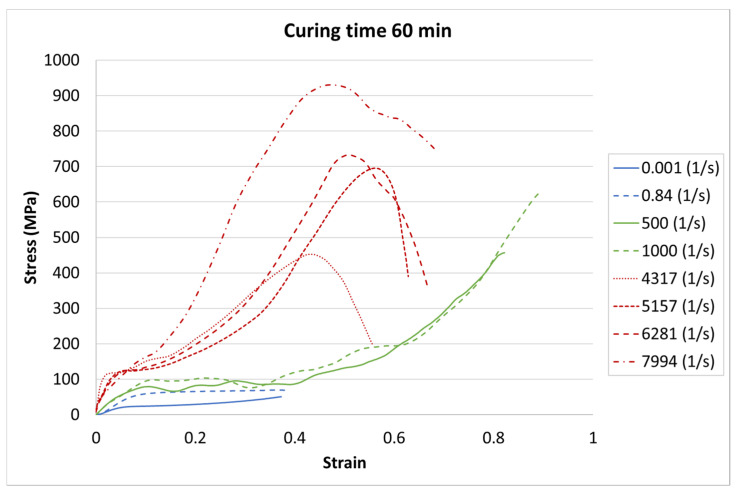
Results of durable resin tested under various strain rates for a curing time of 60 min for UV and heat exposure.

The results for each curing time are presented in Figure 6, Figure 7 and Figure 8.

The achieved results show a strain rate effect dependent on the curing process. When a higher strain rate was applied, the samples showed more rigid behavior. In each case, the same character of curves appeared. The greatest strength appeared for the highest strain rate value of 7994 1/s, whereas the weakest strength was in the static test with a strain rate of 0.001 1/s.

The next step of the results analysis was the comparison of the test results for each strain rate value for different curing times. The comparison is presented in Figure 9, Figure 10, Figure 11, Figure 12, Figure 13, Figure 14, Figure 15 and Figure 16.

The results presented above show that the curing time influences the strain behavior of the tested resin in each strain rate. For the static tests (0.001 and 0.85 1/s), this effect is clearly visible between the 5-min curing time and the 30-min and 60-min curing times. The decrease in the strength parameters is about 15%. It must also be noted that there is almost no difference between the 30-min and the 60-min curing time.

For the dynamic test carried out on the impact hammer, there is little difference between the sample behavior in each curing time. Such a situation can be caused by the specific dynamic impact of the machine. The mechanism response to the test can cause the differences for each case to be negligible.

For the SHPB test, there is a clear difference for the various curing times. As in the static tests, the biggest difference is between 5-min and the other curing times. However, there were some dynamic effects that caused the irregularity in the response pattern. For example, for the 6281 1/s strain rate, the sample cured in 5 min achieved the highest stress value. For the strain rate of 7994 1/s, the stiffest sample was the one cured for 30 min. Such a difference can be caused by the vibration of rods or other dynamic behavior of the testing machine.

Two kinds of strengthening can be noticed. First, the tested material strengthened with the strain rate increase, which is a well-known phenomenon for polymers [32,33]. Second, material strengthening appeared with the curing time increase. Here, it must be noted that the power of the UV laser light used for sample development was limited. Too high a power during photopolymerization in a 3D printer also causes the hardening of the resin around the focused laser spot. Because of the laser power limitation, post-curing is required. The tested material was not fully hardened after 5 min of the post-curing process.

When analyzing the above data, it must be mentioned that the presented experimental results are shown as representative charts so that the results are clear. All data were analyzed statistically in accordance with the following formulations.

- average value:

(3) $$\bar{x}=\frac{1}{n}\overset{n}{\underset{i=1}{\sum }}x_{i}$$

- standard deviation:

(4) $$s=\sqrt{\frac{1}{n-1}\overset{n}{\underset{i=1}{\sum }}{\left(x_{i}-\bar{x}\right)}^{2}}$$

- double-sided confidence interval:

(5) $$\bar{x}-\frac{t_{0.95}}{\sqrt{n}}s<m<\bar{x}+\frac{t_{0.95}}{\sqrt{n}}s$$

where *x_i_* is the value of the *i*-th measurement, *n* is the number of all measurements, and *t_0.95_* is the quantile of Student’s t-distribution for the given volume of measurements.

For all the performed tests, the analyzed parameter was the stress value at the 0.05 strain, which was closely related to the initial course of the stress-strain diagram.

The data achieved from statistical analysis are presented in Table 2, Table 3 and Table 4. In the tables, *x_n_* is an updated average of stress at a strain of 0.05.

**Figure 17:**
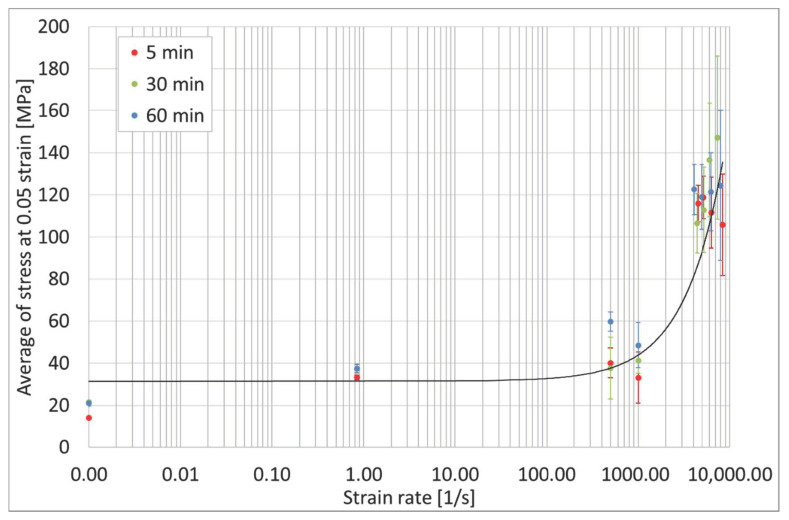
Statistical analysis of results for an average value of stress under a 0.05 strain vs. a strain rate.

In addition, the average value of stress under the 0.05 strain vs. strain rate is presented as a chart in Figure 17.

Based on the presented statistical analysis, it can be concluded that, for the static tests especially, the length of the post-curing time had the greatest effect with a very small standard deviation. This difference is significant for the samples with the shortest curing time compared to the samples cured in 30 min and 60 min. The mechanical properties of samples exposed to longer times differ slightly, so it can be assumed that the exposure of samples exceeding 30 min slightly changes the strength parameters of the tested material. For the dynamic tests, similar dependencies can be seen, but the analyzed values are characterized by a greater standard deviation, which proves a lower repeatability and accuracy of the results. Tests at very high strain rates were associated with a much greater level of difficulty in execution, recording, and signal analyzing. In general, the tested material was characterized by the highest stress values at the 0.05 strain, but, due to the wide-ranging results, it is not possible to clearly state the influence of exposure time on the mechanical properties.

## 5. Conclusions

To summarize, compression tests of photocurable durable resin with different strain rate values have been shown. The tests were carried out in static, dynamic, and SHPB conditions. In addition, the time of the curing process (UV exposure and heating) was considered. The samples showed differences in the mechanical response in accordance with the applied strain rate and the curing time.

The strengthening of the photocurable resin appeared when a higher strain rate was applied. The character of the stress-strain curves was the same for the static, dynamic, and SHPB tests. The greatest value of stress was noticed for the highest strain rate value of 7994 1/s in the SHPB test. The smallest value appeared in the static test (0.001 1/s).

The curing time also influenced the strengthening process for each strain rate considered in the paper. For the static and dynamic tests, the difference in the mechanical parameters is significant for the samples with the shortest curing time of 5 min. For the times of 30 and 60 min, the change in properties is not as significant. It was also noticed that the dynamic test results were characterized with lower repeatability and accuracy.

The tests carried out with the use of the SHPB (the highest strain rates) gave the results in which the curing time effect was not clearly visible. The material was characterized by the highest stress values in those tests, but the strict rule as in the static and dynamic studies was not drawn.

All the achieved results were statistically analyzed. The considered parameter was the stress value at a 0.05 strain.

The presented results can be used in the real structures’ designing process and for the finite element modelling [34,35]. The study on the mechanical properties of such material considering the strain rate effect is desired in the energy absorption applications, impact constructions, or blast protective mechanisms.

## Figures and Tables

**Figure 9:**
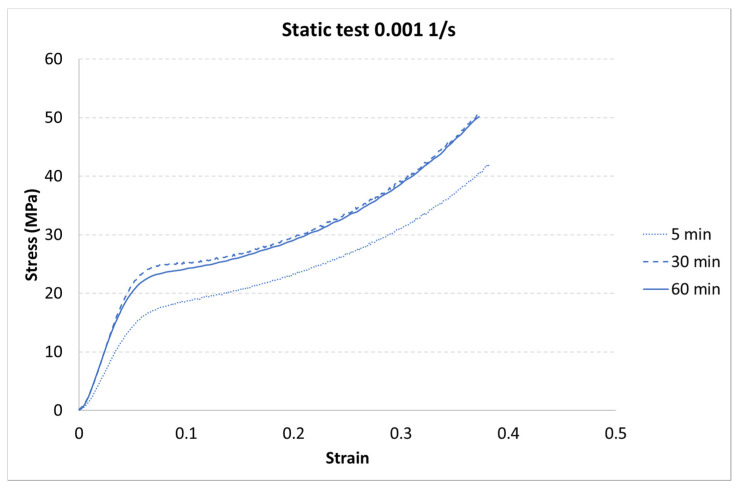
Results of the static test (strain rate of 0.001 1/s) of durable resin for curing times of 5, 30, and 60 min for UV and heat exposure.

**Figure 10:**
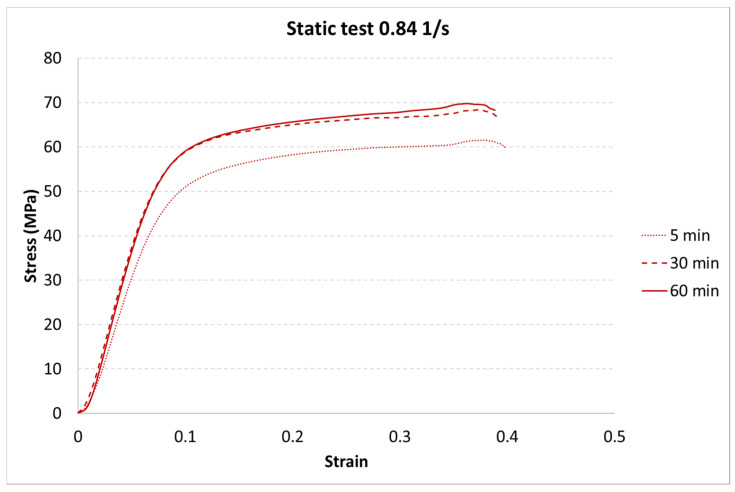
Results of the static test (strain rate of 0.84 1/s) of durable resin for curing times of 5, 30, and 60 min for UV and heat exposure.

**Figure 11:**
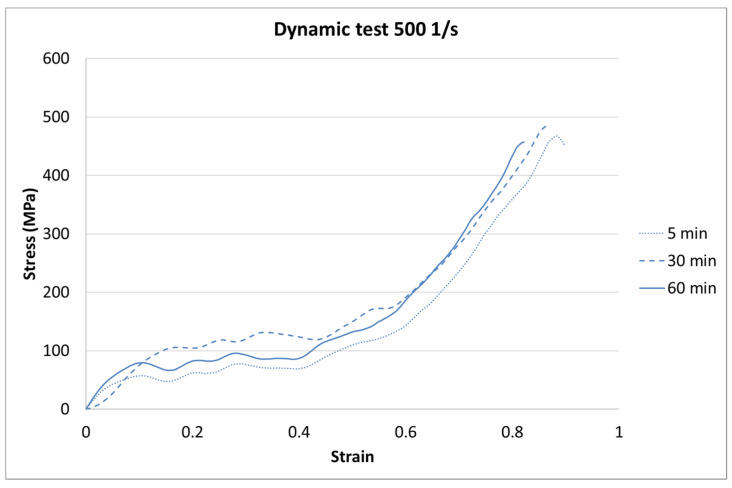
Results of the dynamic test (strain rate of 500 1/s) of durable resin for curing times of 5, 30, and 60 min for UV and heat exposure.

**Figure 12:**
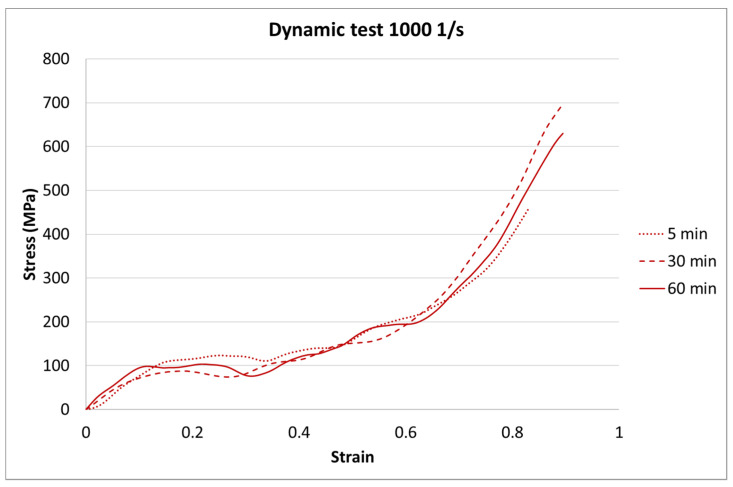
Results of the dynamic test (strain rate of 1000 1/s) of durable resin for curing times of 5, 30, and 60 min for UV and heat exposure.

**Figure 13:**
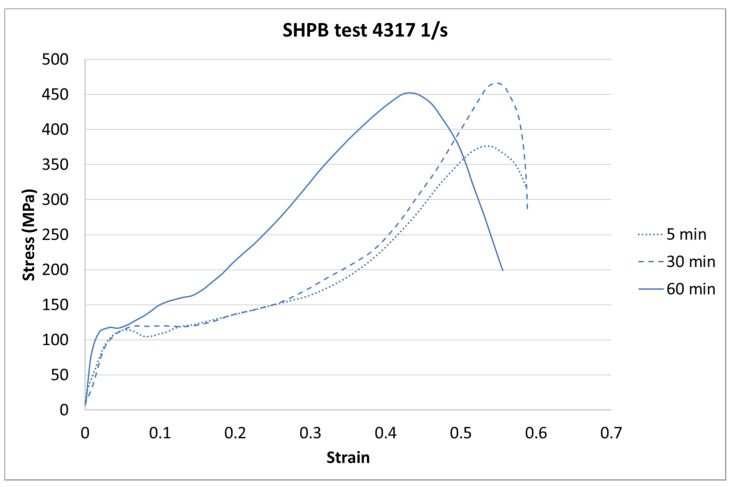
Results of the Split Hopkinson’s Pressure Bar (SHPB) test (strain rate of 4317 1/s) of durable resin for curing times of 5, 30, and 60 min for ultraviolet (UV) and heat exposure.

**Figure 14:**
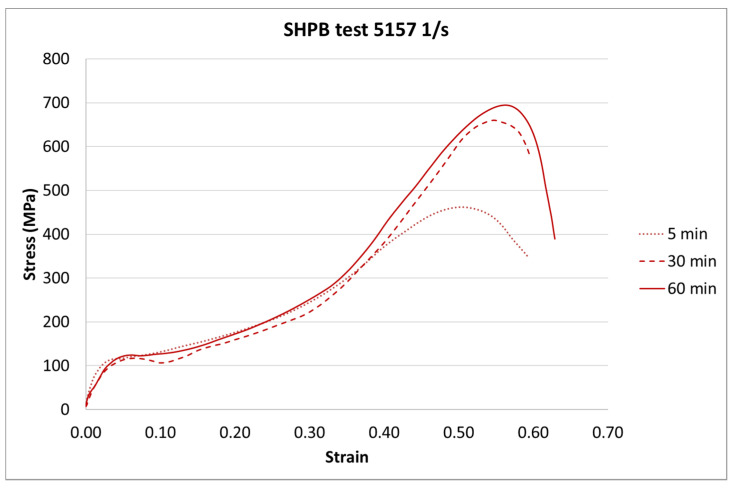
Results of the SHPB test (strain rate of 5157 1/s) of durable resin for curing times of 5, 30, and 60 min for UV and heat exposure.

**Figure 15:**
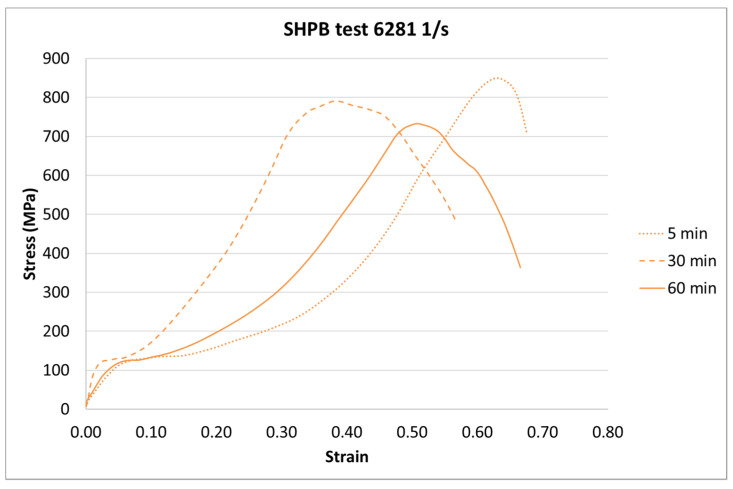
Results of the SHPB test (strain rate of 6281 1/s) of durable resin for curing times of 5, 30, and 60 min for UV and heat exposure.

**Figure 16:**
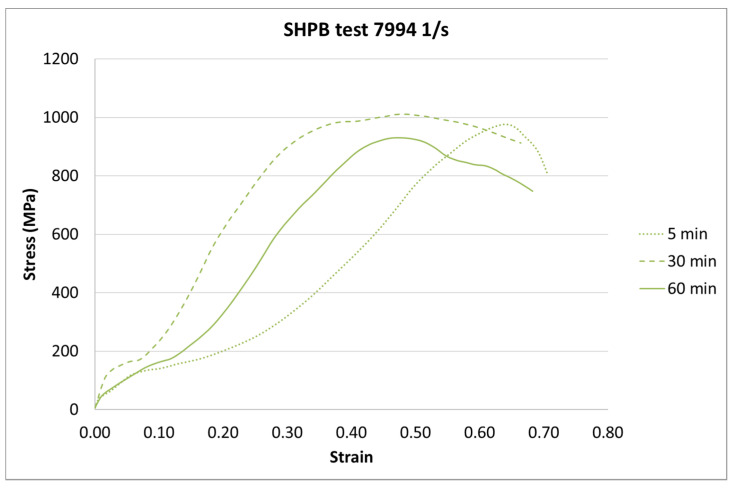
Results of the SHPB test (strain rate of 7994 1/s) of durable resin for curing times of 5, 30, and 60 min for UV and heat exposure.

**Table 1:** Samples dimensions and achieved strain rates.

Test	Sample Height (mm)	Sample Diameter (mm)	Strain Rate (1/s)
Static	5	6	0.001
	5	6	0.84
Dynamic	3	12	500
	3	12	1000
SHPB	2.5	12	4317
	2	12	5157
	1.5	12	6281
	1	12	7994

**Table 2:** Statistical analysis results for the static tests.

	Stress at the 0.05 Strain (MPa)							
Strain Rate and Curing Time	1	2	3	$\mathrm{Average}\;\mathrm{Value},\;\bar{x}$	Standard Deviation, *s*	*x*_1_	*x*_2_	*x_n_*
0.001 1/s, 5 min	**14.15**	**13.66**	**14.76**	14.19	0.55	13.26	15.12	14.19
0.84 1/s, 5 min	**32.72**	**34.45**	**32.62**	33.26	1.03	31.53	35.00	33.26
0.001 1/s, 30 min	**21.78**	**21.46**	**21.1**	21.45	0.34	20.87	22.02	21.45
0.84 1/s, 30 min	**34.91**	**38.47**	**38.55**	37.31	2.08	33.81	40.81	37.31
0.001 1/s, 60 min	**20.27**	**20.18**	**22.67**	21.04	1.41	18.66	23.42	21.04
0.84 1/s, 60 min	**38.7**	**38.6**	**35.09**	37.46	2.06	34.00	40.93	37.46

selected values marked by bold.

**Table 3:** Statistical analysis results for the dynamic tests.

	Stress at the 0.05 Strain (MPa)									
Strain Rate and Curing Time	1	2	3	4	5	$\mathrm{Average}\;\mathrm{Value},\;\bar{x}$	Standard Deviation, *s*	*x*_1_	*x*_2_	*x_n_*
500 1/s, 5 min	31.47	**42.43**	**34.30**	**43.43**	**49.00**	40.13	7.14	33.32	46.93	40.05
1000 1/s, 5 min	**35.13**	14.40	**34.82**	**33.69**	**48.02**	33.21	12.04	21.73	44.69	34.55
500 1/s, 30 min	52.65	**31.70**	**24.87**	**24.58**	53.88	37.54	14.64	23.57	51.50	27.05
1000 1/s, 30 min	**36.25**	34.34	**41.05**	**48.34**	46.65	41.33	6.17	35.45	47.21	41.32
500 1/s, 60 min	67.59	**58.51**	**59.44**	**56.62**	**56.76**	59.78	4.52	55.47	64.10	57.83
1000 1/s, 60 min	**56.06**	**43.69**	**55.44**	**55.60**	31.79	48.52	10.70	38.31	58.72	52.70

selected values marked by bold.

**Table 4:** Statistical analysis results for the SHPB tests.

	Stress at 0.05 Strain (MPa)														
Strain Rate and Curing Time	1	2	3	4	5	6	7	8	9	10	$\mathrm{Average}\;\mathrm{Value},\;\bar{x}$	Standard Deviation, *s*	*x*_1_	*x*_2_	*x_n_*
4317 1/s, 5 min	135.06	107.48	**111.98**	**112.48**	108.75	124.82	**113.55**	**117.08**	108.02	**119.90**	115.91	8.70	110.87	120.95	115.00
5157 1/s, 5 min	134.19	**115.13**	128.33	**123.11**	**113.62**	**126.15**	**113.17**	97.49	**117.61**	**119.64**	118.84	10.16	112.96	124.73	118.35
6281 1/s, 5 min	137.51	**118.77**	**115.36**	**109.75**	91.62	**111.49**	77.06	**118.37**	123.94	**111.52**	111.54	16.77	101.82	121.26	114.21
7994 1/s, 5 min	**111.24**	142.22	88.40	137.11	**110.35**	79.29	**118.02**	64.71	**104.44**	**101.57**	105.74	24.08	91.77	119.70	109.12
4317 1/s, 30 min	**112.54**	**112.62**	**106.46**	**112.99**	116.07	68.38	**113.85**	**109.68**	**101.07**	**112.26**	106.59	14.09	98.42	114.76	110.18
5157 1/s, 30 min	62.97	**117.21**	**109.45**	**123.52**	**113.92**	**114.36**	**123.36**	**107.41**	143.28	**113.71**	112.92	20.28	101.16	124.68	115.37
6281 1/s, 30 min	**127.56**	**133.79**	**130.44**	99.42	202.48	**147.12**	**133.32**	115.07	**131.09**	**145.00**	136.53	26.95	120.91	152.15	135.47
7994 1/s, 30 min	**154.12**	77.44	**160.67**	124.27	**162.50**	**132.01**	**133.15**	**127.07**	224.82	176.17	147.22	38.81	124.73	169.72	144.92
4317 1/s, 60 min	112.10	141.12	134.27	**117.08**	**124.37**	140.21	108.56	**118.96**	**116.79**	112.58	122.60	11.91	115.70	129.51	119.30
5157 1/s, 60 min	**112.70**	**119.86**	129.19	**114.79**	148.56	100.46	128.16	93.39	**121.52**	**122.49**	119.11	15.41	110.18	128.05	118.27
6281 1/s, 60 min	134.95	142.07	86.42	132.45	**118.22**	99.49	**119.00**	**122.36**	**114.54**	145.25	121.48	18.47	110.77	132.18	118.53
7994 1/s, 60 min	**133.98**	199.46	**107.83**	93.79	154.40	87.39	**135.92**	**119.86**	79.28	**132.34**	124.43	35.68	103.74	145.11	125.99

selected values marked by bold.
